# Single graphene nanoplatelets: capacitance, potential of zero charge and diffusion coefficient†
†Electronic supplementary information (ESI) available. See DOI: 10.1039/c5sc00623f



**DOI:** 10.1039/c5sc00623f

**Published:** 2015-03-04

**Authors:** Jeffrey Poon, Christopher Batchelor-McAuley, Kristina Tschulik, Richard G. Compton

**Affiliations:** a Department of Chemistry, Physical and Theoretical Chemistry Laboratory , University of Oxford , South Parks Road , Oxford , OX1 3QZ , UK . Email: richard.compton@chem.ox.ac.uk

## Abstract

Nano-impact chronoamperometric experiments are a powerful technique for simultaneously probing both the potential of zero charge (PZC) and the diffusion coefficient (*D*_0_) of graphene nanoplatelets (GNPs). The method provides an efficient general approach to material characterisation. Using nano-impact experiments, capacitative impacts can be seen for graphene nanoplatelets of 15 μm width and 6–8 nm thickness. The current transient features seen allow the determination of the PZC of the graphene nanoplatelet in PBS buffer as –0.14 ± 0.03 V (*vs.* saturated calomel electrode). The diffusion coefficient in the same aqueous medium, isotonic with many biological conditions, for the graphene nanoplatelets is experimentally found to be 2 ± 0.8 × 10^–13^ m^2^ s^–1^. This quick characterisation technique may significantly assist the application of graphene nanoplatelets, or similar nano-materials, in electronic, sensor, and clinical medicinal technologies.

## Abstract 2

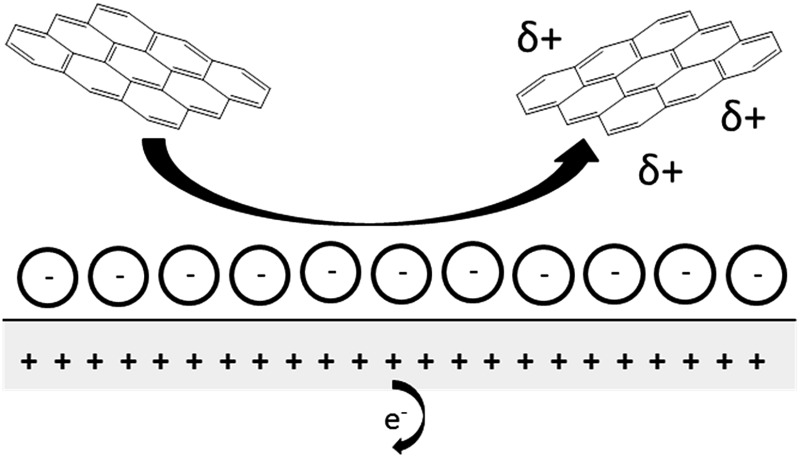
A nano-impact chronoamperometric experiment is presented here as a powerful technique for simultaneously probing important physical properties of graphene nanomaterials.

## 1. Introduction

Carbon materials have widespread applications in modern technologies. Since the discovery of graphene and subsequent groundbreaking experiments in 2004,^1^ for which both Geim and Novoselov received the 2010 Nobel Prize in Physics, the interest in graphene and related carbon materials has exploded in recent years. From 2006 onwards, much literature has been published exploring a wide range of possibilities for the application of such materials.^2–4^ The enhanced electrical and thermal conductivity, large surface area, higher charge mobility and carrier concentration, and mechanical strength of graphene materials allows their many applications in prototypes within sensing and energy storage technologies.^5–8^ A broad range of possible technological applications vary from the manufacturing of supercapacitors,^9–15^ dye-sensitised solar cells,^16,17^ biological molecule sensors,^18,19^ and catalyst supports,^20^ to the field of nano-medicine using pristine graphene or graphene materials.^21,22^ However, the large scale manufacture of pristine graphene through graphite exfoliation or reduced graphene oxide for industrial applications remains expensive.^23^ Therefore the use of existing cheaper graphene derivatives and incorporation into composites is an attractive alternative.^24^ Graphene nanoplatelets (GNPs) belong to this family of graphene materials, where possible applications are currently being intensely investigated.^20,25–28^ GNPs enjoy the advantageous properties of graphene and highly ordered graphitic materials but avoid the poor stability of graphene;^29^ whereby graphene is susceptible to structural distortion in that sheets around 1 nm in width show spontaneous ripping.^30^ Current research focuses on the usefulness of GNPs in electronics. Hence it is important to gain further understanding of the capacitative properties of GNPs. In particular, the GNPs' potential of zero charge (PZC) is a very important parameter in determining the nature of the electrode–electrolyte interface of a particular electrochemical system.^31^

It has been reported that GNPs are suitable for electrochemical detection and removal of endocrine-disrupting chemicals,^32^ nanocomposite cancer sensors,^33^ nanoparticle decorated cholesterol sensors,^34^ and detectors of biomarkers to name a few.^35^ It is therefore essential to understand the mass transport properties of graphene materials to assist their application in biotechnology and medicine. This aspect of the GNPs needs to be well characterised prior to clinical application.

**Fig. 1:**
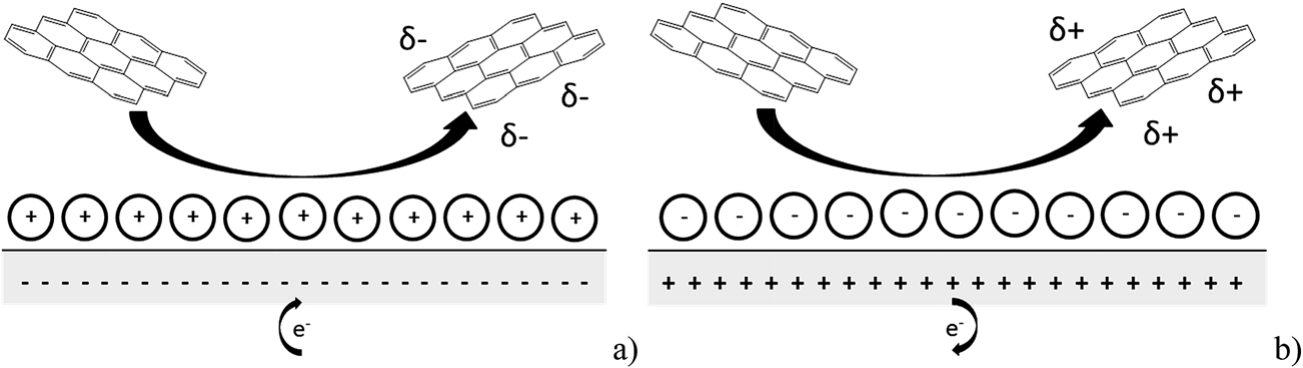
Schematic diagram of the process postulated to occur during GNP impact where (a) the electrode is negative with respect to the PZC and (b) the electrode is positive with respect to the PZC.

In this paper we report the use of nano-impact experiments to obtain the PZC and the diffusion coefficient of GNPs.^36^ The GNPs impact the cylindrical carbon fibre wire microelectrode in a stochastic manner through Brownian motion. The GNP's collision with the potentiostated electrode removes charge from the electrode–electrolyte interface. To maintain charge neutrality, electrons enter or leave the electrode when the applied potential on the electrode is respectively positive or negative with respect to the PZC. The work herein demonstrates the nature of the current transients, caused by the aforementioned electron transfers, seen in nano-impact experiments to be capacitative charging in nature, with a schematic diagram shown in Fig. 1.^37^

The use of this experiment can also be extended to the general characterisation of nanocomposite materials of industrial interest. In the past, the diffusion coefficient and the PZC of a material have had to be investigated individually in separate experiments.^38^ This paper aims to show that nano-impact chronoamperometric experiments are an easy and powerful technique to simultaneously probe physical properties such as the PZC and the diffusion coefficient (*D*_0_) of the nanoplatelets. This provides an efficient general method for material characterisation.

## 2. Experimental

### 2.1 Chemicals and reagents

All chronoamperometric measurements were carried out in a supporting electrolyte of 0.1 M potassium chloride, 50 mM potassium monophosphate, 50 mM potassium diphosphate (PBS) buffer solution at pH = 6.8. All reagents were provided by Sigma-Aldrich at reagent grade unless stated otherwise. The graphene nanoplatelets (GNPs, 15 μm wide, 6–8 nm thick) were purchased from Strem Chemicals, MA, USA. All reagents were used without further purification. All solutions were prepared with deionised water of resistivity not less than 18.2 MΩ cm at 298 K (Millipore, Billerica, MA). All electrolytes were degassed with pure nitrogen gas for 5 min.

**Fig. 2:**
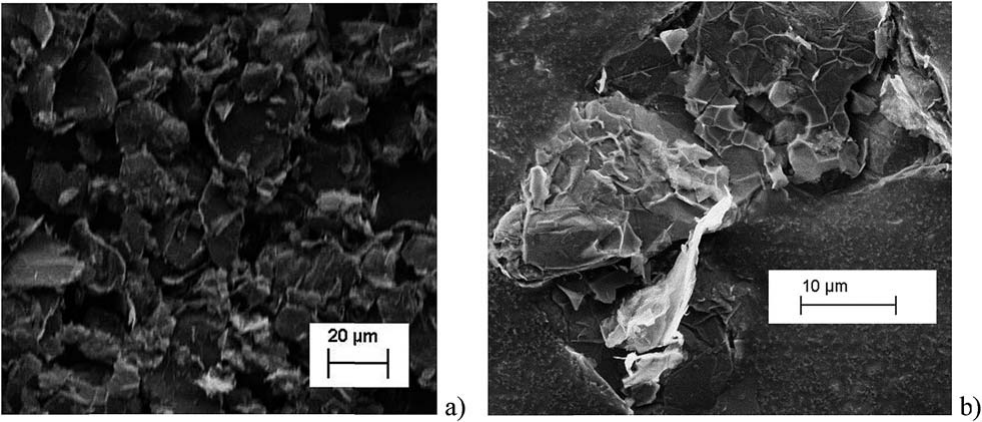
SEM images of the graphene platelets used in this work.

Imaging of the graphene nanoplatelets was performed by Scanning Electron Microscopy (SEM) employing a Leo Gemini II Field Emission Gun Scanning Electron Microscope (Zeiss, Oberkochen, Germany) using an acceleration voltage of 5 kV and a detector in in-lens geometry. The GNP powder was immobilised on a SEM sample holder using adhesive carbon tape. To reduce electrical charging during the measurement, a thin layer of gold was sputtered (Cressington sputter coater 108 auto) on top of the sample. Representative SEM images are shown in Fig. 2 and are analysed using the software ImageJ developed by the National Institutes of Health, BD, US, to determine the size distribution of the GNPs. In Fig. 2, platelet features were clearly seen, many rectangular or triangular in shape with widths close to the specified 15 μm by Strem.

**Fig. 3:**
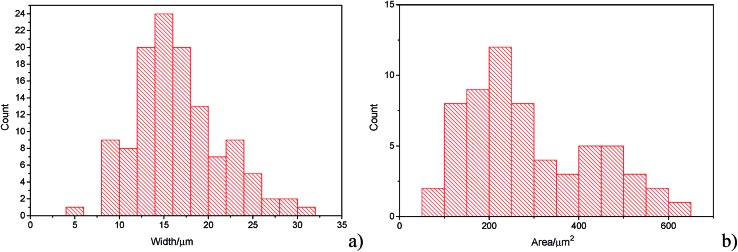
(a) Histogram of widths of graphene nanoplatelets (b) histogram of area (assuming nanoparticles as rectangular platelets).

The histograms (Fig. 3a and b) show a good agreement with the quoted width value of 15 μm from the supplier Strem Chemicals. From the SEM image analysis, the average width is 16.5 ± 5 μm (sample population = 120, bin size = 2 μm), whereas the average GNP area is 297 ± 152 μm^2^ (sample population = 62, bin size = 50 μm^2^). The thickness of graphene nanoplatelets is 7.1 ± 2 nm (see Fig. S1 in the ESI†), in concordance with Strem's specification sheet.^39^ Using the experimentally derived thickness of 0.37 nm/graphene layer measured by Koh *et al.* ^40^ (*cf.* 0.34 nm/layer in graphite),^41^ each GNP consists of *ca.* 16–22 graphene layers.^42^

### 2.2 Suspension preparation

A 1.2 × 10^–12^ mol dm^–3^ GNP suspension was prepared by adding 11.0 mg of GNPs to 100 mL of the PBS supporting electrolyte mentioned above, assuming a relative molecular mass of 9.3 × 10^10^ g mol^–1^ for GNPs calculated from the 1 × 10^5^ g m^–3^ bulk density provided from Strem. The suspension was sonicated using a Fisher Scientific FB15050 ultrasonic bath for 15 seconds to disperse the nanoplatelets. Suspensions of lower concentrations (1.2 × 10^–14^ mol dm^–3^, 3.5 × 10^–14^ mol dm^–3^, 5.9 × 10^–14^ mol dm^–3^, and 1.2 × 10^–13^ mol dm^–3^) were derived from this suspension without any further sonication. In order to ensure a homogeneous suspension of GNPs in solution, the suspension was vigorously shaken before any dilution or transfer into other vessels.

To prepare the 5.9 × 10^–13^ mol dm^–3^ GNP suspension, 5.5 mg of GNPs was added to 100 mL of supporting electrolyte. The suspension was likewise sonicated for 15 seconds once per preparation in order to break up any aggregated powder GNP lumps for a better dispersed suspension.

### 2.3 Carbon fibre wire microelectrode fabrication

The method of cylindrical carbon fibre wire microelectrode fabrication follows that reported by Ellison *et al.* ^43^ First, a 7.0 μm diameter carbon fibre (Goodfellow Cambridge Ltd.) was connected to a metal wire using silver epoxy (RS Components Ltd.) conductive adhesive. The adhesive was set by heat treatment in an oven for 15 min at approximately 60 °C. The wire was then threaded through a plastic micropipette tip. The interstice between the carbon fibre/metal wire and the plastic tip was sealed using cyanoacrylate adhesive, and the wire pulled down so only the carbon fibre was extended out of the end. To ensure the setting of the cyanoacrylate adhesive, the electrode was then left to rest for 12 h. Finally, the carbon fibre tip was cut to a length of approximately 1 mm protrusion past the sealed end.

### 2.4 Electrochemical procedures

Chronoamperometric experiments were recorded using a carbon wire microelectrode (CWM) as a working electrode, a saturated calomel electrode (SCE) as the reference electrode, and a platinum gauze as the counter electrode. Potentiostatic control and measurement of the impact current transients were enabled by the use of the in-house built low noise potentiostat.^44^ This potentiostat comprises three main sections; the computer interface used for analog-to-digital and digital-to-analog conversion, the current amplifier circuit and the stabilised potentiostat. A Labjack U6 (Labjack corporation, Lakewood, CO. USA), with a Labjack tickDAC, was used for the computer interface. Connection to the Labjack was *via* a standard USB but with the ground isolated from that of the PC (USB-ISO OLIMEX, Farnell, Leeds, UK). Control of the Labjack was performed *via* a script written in Python 2.7 and run through the IDE Canopy (Enthought, Austin, TX USA). Measurement of the current at the working electrode (running to ground) was achieved with a low current-amplifier LCA-4K-1G (FEMTO, Messtechnik GmbH, Germany) and the bandwidth of the output of the current amplifier was limited using a 100 Hz 2-pole passive RC filter, Linear Technology DC338A-B (Farnell, Leeds, UK). The resulting analog signal was oversampled and digitised using the Labjack at a stream rate of 4 kHz. Potentiostatic control was provided by a highly stabilised (1 kHz bandwidth) classic adder potentiostat.^45^ Importantly, first, for the reference buffer a high quality operational-amplifier LMC6001 (Farnell, Leeds, UK) with ultra low-input bias (25 fA) was used. Second, a high quality low-noise operational-amplifier, AD797 (Farnell, Leeds, UK), provided the control of the potential at the counter electrode.

### 2.5 Nano-impact experiments

Chronoamperometric experiments were conducted at room temperature for different GNP concentrations and potentials to collect capacitative impact data and to analyse their frequency and the charge transferred per impact event.^36^ GNP suspensions of different particle concentrations were prepared as described above and were purged with pure nitrogen gas for 5 minutes prior to the experiments to ensure the suspensions were well mixed. A CWM was then immediately immersed into the GNP suspension and a constant potential was applied for 20 seconds by stepping the potential from the open circuit potential to the desired value. Ten chronoamperograms were recorded before the suspension was again bubbled with nitrogen gas, these 10 measurements are referred to as ‘a set’ herein. For frequency analysis investigations, the process was repeated until at least 4 ‘sets’ of impact data were collected. Each ‘set’ was obtained within a timeframe of approximately 10 min. During this experimental time no significant sedimentation of GNPs was observed. The suspension is therefore assumed to be stable over the timescale of the experiments. To ensure capacitative impacts had sufficient charge and intensity to be easily identifiable, a high applied potential of +1.20 V was used to study the impact frequency. For investigations concerning capacitative charge with respect to potential applied ranging from +1.20 V to –1.20 V *vs.* SCE, at least 5 scans with detected impacts were collected for each applied potential increment. SignalCounter developed by Dr Dario Omanović (Division for Marine & Environmental Research, Ruder Bošković Institute, Zagreb, Croatia), was used to analyse scans.^46^

## 3. Results and discussion

The following sections report the results observed from the impact experiments conducted in order to elucidate the nature of the current transients (‘impacts’) seen (Section 3.1), the potential of zero charge (PZC) of the GNP (Section 3.2), and the mass transport characteristics of the GNPs (Section 3.3).

First, the origin of current transient features in the presence of GNPs is evidenced to be due to capacitative charging. Second, by varying the potential between +1.20 V and –1.20 V (*vs.* SCE) in a 5.9 × 10^–13^ mol dm^–3^ GNP suspension, impact experiments were utilised to establish the PZC of the graphene nanoplatelets in aqueous media from the capacitative charging transient features seen. The results give insights into the electronic structure of the GNPs. Finally, impact experiments were conducted in suspensions of various GNP concentrations under the applied potential of +1.20 V. Transient features seen in such scans were used to investigate the solution phase mass transport properties of the GNPs.

### 3.1 Capacitative impacts of GNPs

A carbon fibre wire electrode was submerged into a 5.9 × 10^–13^ mol m^–3^ suspension of GNPs (pH 6.8 PBS). The electrode was potentiostated at –1.20 V (*vs.* SCE) for a duration of 20 seconds. Impact features were seen in the conducted chronoamperometric experiments. This section elucidates the nature of the impact features seen and their possible physical origins are established.

**Fig. 4:**
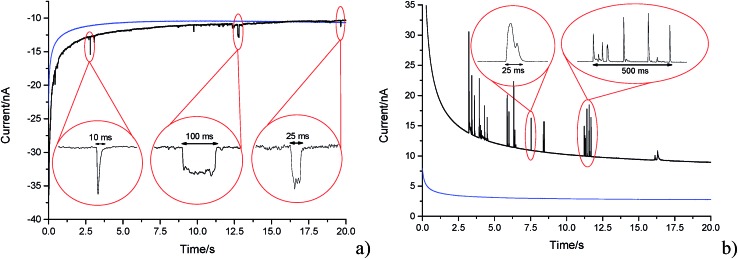
Representative chronoamperometric scans in a 5.9 × 10^–13^ mol dm^–3^ suspension using a cylindrical carbon fibre wire electrode at a potential of (a) –1.20 V (*vs.* SCE); (b) +1.20 V (*vs.* SCE). Blue line: blank scan in 0.1 M KCl with 50 mM KH_2_PO_4_, 50 mM K_2_HPO_4_ (PBS) buffer solution mentioned in the Experimental section. Black line: 5.9 × 10^–13^ mol dm^–3^ graphene nanoplatelet (GNP) suspension scan showing capacitative impacts.

In the presence of the GNPs, short transient current features were observed in the chronoamperogram for the –1.20 V potentiostated microelectrode, as depicted in Fig. 4a. As highlighted, the impact features observed were either in the form of ‘spikes’ (short transient times) or ‘steps’ (long transient times). The impact durations varied from 10 ms to 100 ms, whereas their magnitude ranged from 0.03 nA to 3 nA. The average charge of impacts at an applied potential of –1.20 V was –14.3 ± 4 pC. In the absence of GNPs in solution, no impact features were observed and the background current associated with the charging of the carbon fibre electrode was found to decrease smoothly in a monotonic fashion (as shown in Fig. 4, blue line). Consequently, the transient features can be attributed to the stochastic impacting of GNP at the potentiostated carbon fibre electrode. The mass transport of such GNP impacts will be governed by diffusion of the nanoplatelets,^43^ with the frequency of impacts scaling linearly with respect to concentration. This will be discussed in detail in Section 3.3 below.

Having evidenced that the presence of the short current spikes was related to the GNPs in solution, the physical origin of the charge transfer process was investigated. Two distinct possibilities arise as GNPs collide with the electrode, where the charge transfer may be either faradaic or capacitative. Faradaic impacts show a sharp ‘on–off’ behaviour of the charge increase with respect to the potential.^47–49^ Capacitative spikes show a steady decrease of spike charge when the applied potential approaches the PZC.^37,50^ Upon alteration of the potentiostatic potential from –1.20 V to +1.20 V (*vs.* SCE) the polarity of the transient GNP spikes were found to change (Fig. 4b). This phenomenon is consistent with the impacting particle removing charge from the electrode–electrolyte interface, as expected of a capacitative impact.^37,50^

To further confirm that the impacts seen are of capacitative origin, it is important to be sure that the oxygen species on the GNPs do not significantly contribute to the impact charge faradaically. The surface oxygen species of the used GNPs (ether, carboxyl, hydroxyl, quinone functionality) amount to a total oxygen content of less than 1% and a residual acid content of less than 0.5% by weight.^39^ The lack of detectable redox activity of surface functionalities of the used GNP in PBS was evidenced by cyclic voltammetric studies using a GNP modified glassy carbon macroelectrode (BAS Technicol, USA, diameter 3 mm) with 7.9 μg of GNPs. As depicted in Fig. S3 (see ESI†), no redox activity in voltammograms was detected for either oxidative or reductive potentials. This is in stark contrast to the inherent electroactivity seen in colloidal graphene oxides.^51^ This confirms the faradaic electrochemical inertness of the oxygen functionalities of GNPs and hence rather suggests the capacitative nature of the impact spikes observed.

**Fig. 5:**
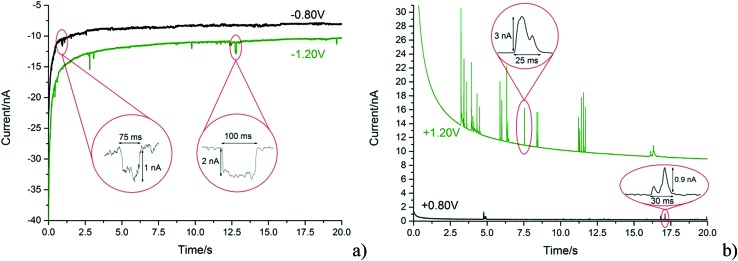
Comparison of spike sizes at different potentials from representative chronoamperometric scans in a 5.9 × 10^–13^ mol dm^–3^ suspension using a cylindrical carbon fibre wire electrode. Smaller features at lower applied potential (*vs.* SCE) magnitude can be seen. (a) Black line: –0.80 V (*vs.* SCE). Green line: –1.20 V (*vs.* SCE). (b) Black line: +0.80 V (*vs.* SCE). Green line: +1.20 V (*vs.* SCE).

With the steadily decreasing magnitude of the applied potential, the impacting particle removes less charge and this results in a steady decrease of spike charge for capacitative impacts.^37^ This was detected as a decrease in spike area and observed as depicted in Fig. 5a and b. In the following section the GNP impacts are studied as a function of potential in the range of –1.20 to +1.20 V (*vs.* SCE), which will be demonstrated to provide a route to determine the potential of zero charge of the graphene material.

### 3.2 PZC determination

The potential of zero charge (PZC) is the potential at which the electrode surface has no excess charge when in contact with an electrolyte.^31,52^ Impact experiments at different potentials can be used to determine the point of applied potential where GNP impacts impart no current transients from capacitative charging. This is the PZC point due to the lack of a double layer to provide charge from the electrode–electrolyte interface upon GNP impacts.^37^ The parameter has significant implications on information concerning the structure of the electrode–solution interface and the electrical double layer (EDL) effects on electrode kinetics.^53,54^

**Fig. 6:**
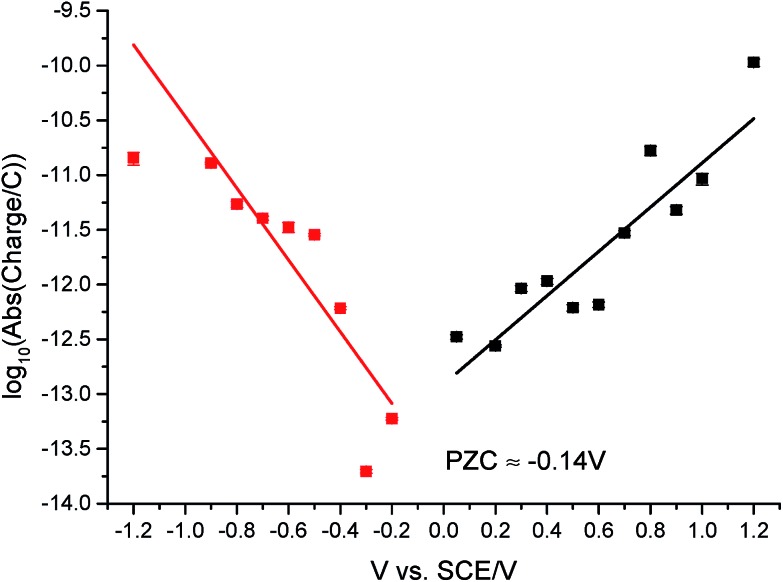
5.9 × 10^–13^ mol dm^–3^ suspension impacts with absolute charge of nanoparticle impacts in logarithmic (log_10_) scale plotted against applied potential (*vs.* SCE). Red squares: values with applied potential < 0 V; black squares: values with applied potential > 0 V (*vs.* SCE). The intersection potential of the two lines is used to estimate the PZC value of GNPs, where PZC is determined to be –0.14 V (*vs.* SCE).

In this section, the capacitative impacts seen in Section 3.1 are used to determine the PZC of the GNPs. To determine the PZC, experiments varying the electrode potential between –1.20 V and +1.20 V (*vs.* SCE) with a GNP concentration of 5.9 × 10^–13^ mol dm^–3^ were conducted. The polarity of the spikes changes as a function of potential. This concurs with the observation that the spikes are caused by capacitative charging, shown in Fig. 1 and 4. Capacitative impacts were seen in each applied potential increment. The magnitude of the resultant impacts was investigated. From Fig. S2,† the increase of average impact charge with respect to the increase of applied potential magnitude away from the PZC is non-linear; a logarithmic plot confirms this non-linear relationship. In Fig. 4, spikes at potentials negative of the PZC have negative polarity. Fig. 6 shows the logarithm (log_10_) of the *absolute* mean integrated charge passed during an impact event as a function of the applied potential. The red and black squares represent reductive and oxidative spike charges respectively. In Fig. 6, linear fittings can be seen for both oxidative and reductive capacitative impacts, implying an approximate logarithmic relationship between average impact charge to applied potential. The significance for GNP capacitance contribution is discussed below. By interpolating the reductive and oxidative fitted lines, these linear fittings provide a convenient tool for the deduction of the point of intersection. At this point of intersection is the PZC, where the impacts result in no transient features in capacitative charging. This point is found to be –0.14 ± 0.03 V (*vs.* SCE).

4 ‘sets’ of chronoamperometric scans per applied potential increment, with a ‘set’ consisting of 10 scans each, were conducted to measure the average impact frequency. A varying number of impacts were seen per scan. The impact frequencies were averaged over each set. For example, at +1.2 V (*vs.* SCE) the average frequency is 0.8 ± 0.2 Hz. The lack of a trend of the average frequency variation with respect to the applied potential is shown in Fig. S4.† Hence there is no apparent frequency bias towards any potential. This demonstrates that despite their smaller capacitative impacts, the experiment is able to observe the smaller sized GNPs at potentials close to the PZC, and they contribute to the impact frequency and charge data as much as the larger GNPs. In other words, despite lower charge impacts and at potentials relatively closer to the PZC, the smaller GNP impacts are not obscured in the background noise and are included into the data collected. This strongly suggests that the full spectrum of GNP sizes is observed and the capacitative impacts charge data are not biased towards larger sized GNPs.

To compare the electronic properties of GNPs with other carbon materials, it is useful to estimate the capacitance of GNPs. The capacitance calculations are performed as such:1
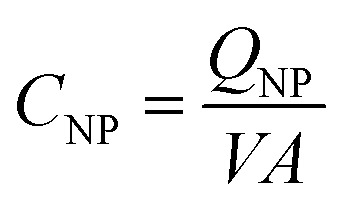



*C*_NP_ is the calculated capacitance of GNPs, *Q*_NP_ is the experimental impact charge, *V* is the applied potential with respect to the estimated PZC, and *A* is the calculated area from the specified dimensions of a GNP (4.5 × 10^–6^ cm^–2^). The values are scaled to the appropriate unit of μF cm^–2^ for ease of comparison. Capacitance values of GNPs are within a range of between 0 to 10 μF cm^–2^. It can be shown in Fig. S6† that the GNPs have a capacitance of the same order of magnitude as that calculated for graphene and similar carbon materials.^55,56^

The section above demonstrates impact experiments to be a viable method to probe the capacitance properties of materials. While the Gouy–Chapman theory predicts a non-linear response of nanoparticle capacitance with applied potential, it is limited to a short range of ±300 mV around the PZC.^57,58^ The wide range of potentials applied experimentally is beyond such a limit. This non-linear relationship instead implies a capacitance contribution from the electronic band structure of the GNPs, rather than a pure double layer contribution.^56^ Therefore the determination of the PZC in aqueous media and the logarithmic relationship of nanoparticle capacitance–potential reflect the electronic properties of the GNP material. This provides an alternative approach to gain information on the electronic band structure of GNPs and its non-constant Density of States (DOS) under various applied potentials.^56,59–62^

### 3.3 GNP diffusion coefficient determination

Chronoamperometric experiments were performed in sets of 10 scans, each with the electrode held at +1.20 V under potentiostatic control for 20 seconds. At +1.20 V, the potential was 1.34 V away from the estimated PZC. This produced larger charged impacts to allow more facile counting. Capacitative spikes and steps were seen and counted using suspensions of five GNP concentrations ranging from 1.6 × 10^–14^ to 5.9 × 10^–13^ mol dm^–3^.

By analysing the impact frequency–concentration dependence, capacitative impacts allow the mass transport properties of the GNPs to be estimated. The GNP is assumed to impact on the cylindrical wire electrode in a stochastic manner, transported *via* diffusion. A theoretical estimation of the GNP impact frequency is therefore needed. The diffusion equation towards a micro-cylinder electrode has been approximately solved by Szabo *et al.* to give:^43,63^
2
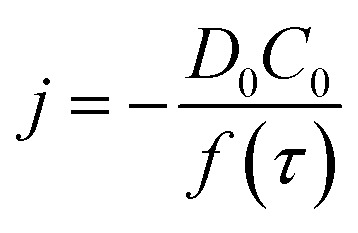

3
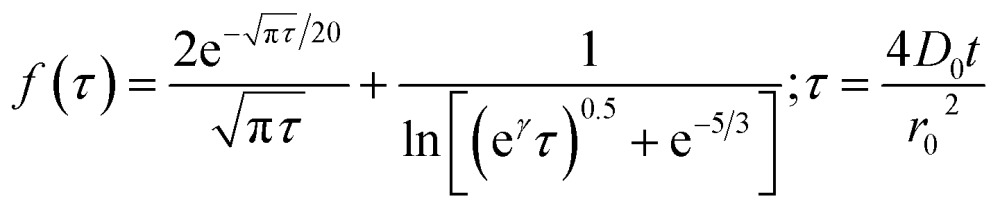
where *j* is the flux to the cylindrical electrode, *D*_0_ is the diffusion coefficient, *C*_0_ is the bulk concentration of the species concerned, the GNPs in this case, *r*_0_ is the electrode radius, and *t* is the duration of the chronoamperometric scan. The *γ* = 0.57721… is a constant derived from the limits of the Bessel functions in the full form of *f*(*τ*). The number of impacts per scan can be derived by integrating eqn (2). The diffusion coefficient of the GNPs is therefore embedded in the flux equation by introducing the definition of *τ* in eqn (3). A theoretical value of the diffusion coefficient is obtained by assuming individual 15 μm wide, as specified by Strem,^39^ square nanoplatelets with an area of 225 μm^2^, to form an infinitesimally thin circular disc, where:4
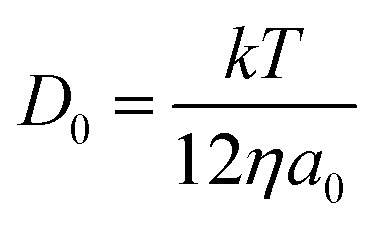

*a*_0_ is the radius of disc and *η* is the viscosity of supporting medium, which for the impact experiments is the PBS buffer mentioned above. The disc is therefore assumed to move in a Stokes–Einstein fashion.^64^

The approximation of the disc as infinitesimally thin is reasonable due to the large aspect ratio between GNP width to thickness (15 μm *vs.* 6–8 nm). However, the non-circular nature of nanoplatelets as illustrated in Fig. 2 may cause deviation from the assumptions mentioned above. The estimated theoretical value is calculated to be around the order of 4 × 10^–14^ m^2^ s^–1^. For example, in a 5.9 × 10^–13^ mol dm^–3^ GNP suspension, the integrated Szabo equation gives an estimated frequency of 0.4 Hz.

**Fig. 7:**
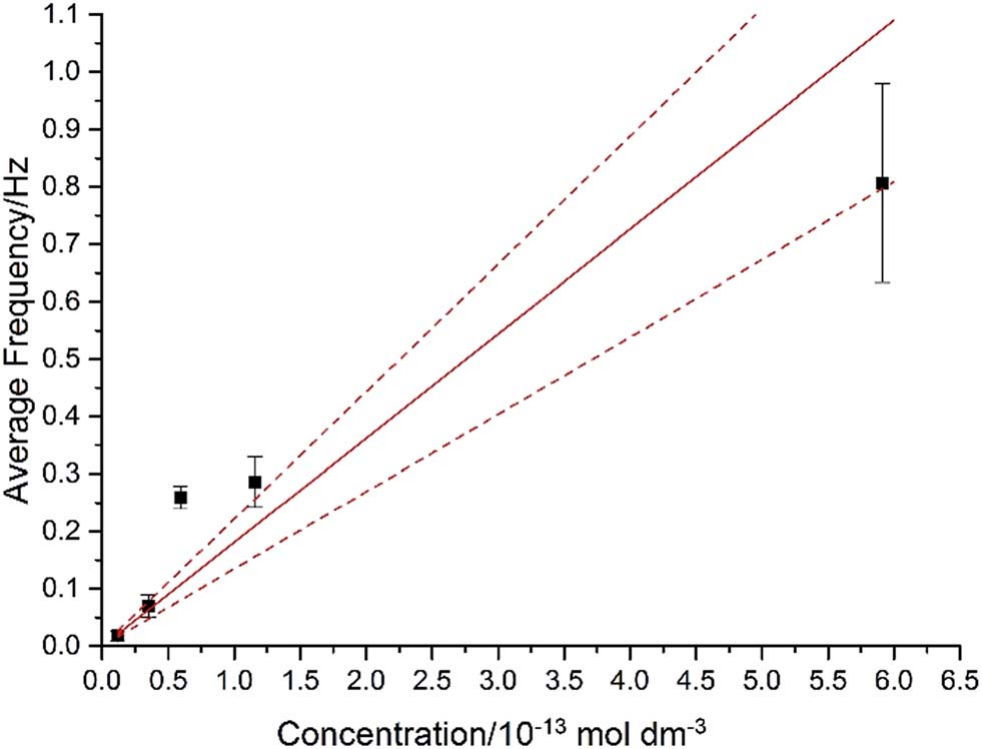
Plot showing the average impact frequency–concentration relationship. The red line plotted indicates a diffusion coefficient of *D*_0_ = 2 ± 0.8 × 10^–13^ m^2^ s^–1^ (error lines dashed). A linear relationship with respect to concentration is predicted by the integrated Szabo equation. All experiments are performed at +1.20 V in order to ensure a large capacitative signal for facile counting.

In Fig. 7, the average impact frequency is plotted against the concentration of GNP suspension. A clear linear fitting is seen. This shows good agreement with the mentioned theory described in eqn (2). The gradient of the fitting estimates the diffusion coefficient of the GNPs to be 2 ± 0.8 × 10^–13^ m^2^ s^–1^. Impacts were seen at 5 different concentrations, from 1.6 × 10^–14^ mol dm^–3^ (4 counts per 10 scans) to 5.9 × 10^–13^ mol dm^–3^ (161 per 10 scans). From the histogram shown in Fig. 3b, the smaller nanoplatelets (area < 225 μm^2^) have a greater contribution to the distribution.^65^ Smaller area nanoplatelets are expected to have a higher diffusion coefficient as described in eqn (4). Therefore it is unsurprising that the experimentally estimated diffusion coefficient (*D*_0_) of the platelets is significantly faster than the calculated theoretical value, as faster diffusing platelets are more likely to impact the electrode than larger GNPs diffusing more slowly.

## 4. Conclusions

Using coulometric impact experiments, capacitative impacts can be seen for graphene nanoplatelets of 15 μm width and 6–8 nm thickness. The current transient features seen allow the determination of the potential of zero charge (PZC) of the graphene nanoplatelets in 0.1 M KCl, 50 mM KH_2_PO_4_, 50 mM K_2_HPO_4_ PBS buffer supporting electrolyte as –0.14 ± 0.03 V. The diffusion coefficient in the same aqueous medium for the graphene nanoplatelets is experimentally found to be 2 ± 0.8 × 10^–13^ m^2^ s^–1^.

In this paper, we have demonstrated that a purely electrochemical method, using nano-impact experiments, is a viable method for determining the PZC of solution phase nanoparticles of graphene. The intersection analysis in Fig. 6 uses capacitative impacts for facile determination of the PZC of nanoparticles in an aqueous suspension, in contrast to other traditional or spectroscopic methods.^66–71^ Amongst others, this is important in light of numerous existing publications suggesting GNP-coated electrodes as a potential electrochemical sensor material, for example, for endocrine-disrupting chemicals.^32^ Coulometric impact experiments using GNPs (15 μm width, 6–8 nm thickness) were demonstrated to enable the first detection of capacitative impacts of these materials at an electrode. The experiments presented herein are conducted in PBS buffer which is isotonic with the human body and many biological organisms. Hence a new and unique route to assess the determination of the PZC and mass transport properties of GNPs in biological conditions has been presented. This may provide useful insights into any potential medicinal, environmental and biological application of GNPs and other nanoparticulate or carbonaceous species.
