# Supplementary material for: Single graphene nanoplatelets: capacitance, potential of zero charge and diffusion coefficient
Source: Chem Sci. 2015 Mar 4;6(5):2869–76. doi: 10.1039/c5sc00623f (PMC5490005; doi:10.1039/c5sc00623f)
Supplement: Supplementary file 1 [file SC-006-C5SC00623F-s001.pdf]

Electronic Supplementary Material (ESI) for Chemical Science.
This journal is © The Royal Society of Chemistry 2015

Single graphene nanoplatelets: capacitance, potential of zero charge and diffusion coefficient

<u>Supporting Information</u>

*Jeffrey Poon, Christopher Batchelor-McAuley, Kristina Tschulik, Richard G. Compton**

Department of Chemistry, Physical and Theoretical Chemistry Laboratory, University of Oxford, South Parks Road, Oxford OX1 3QZ, United Kingdom

*Email: richard.compton@chem.ox.ac.uk

Supporting Information

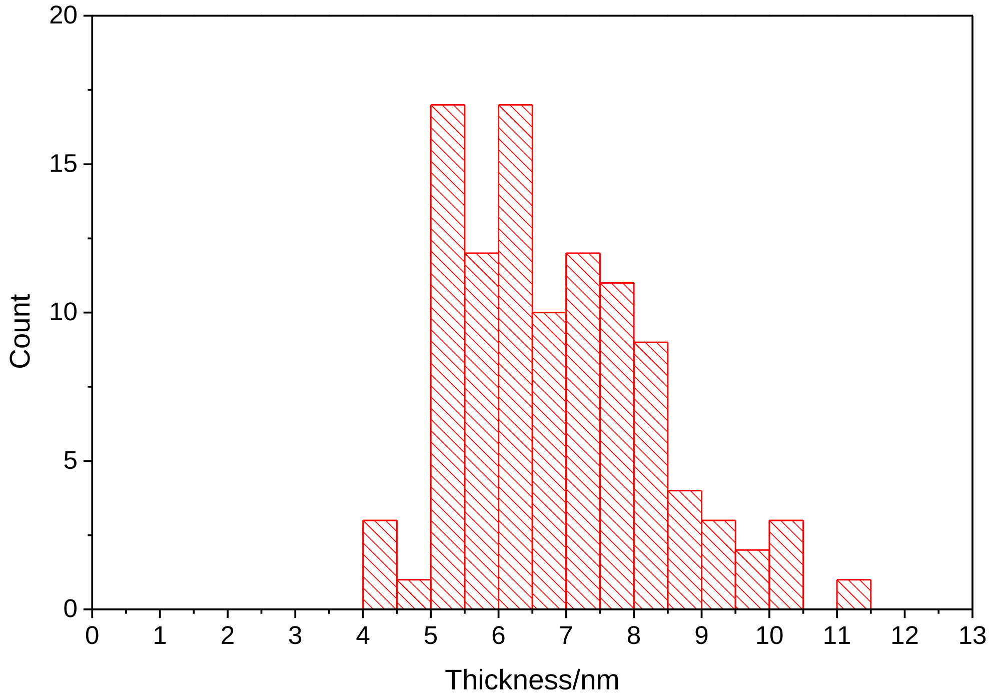

Figure S1: Histogram of thickness of graphene nanoparticles as inferred from SEM analysis with an average thickness of 7.1±2.1 nm. (n = 108, bin size = 0.5 nm)

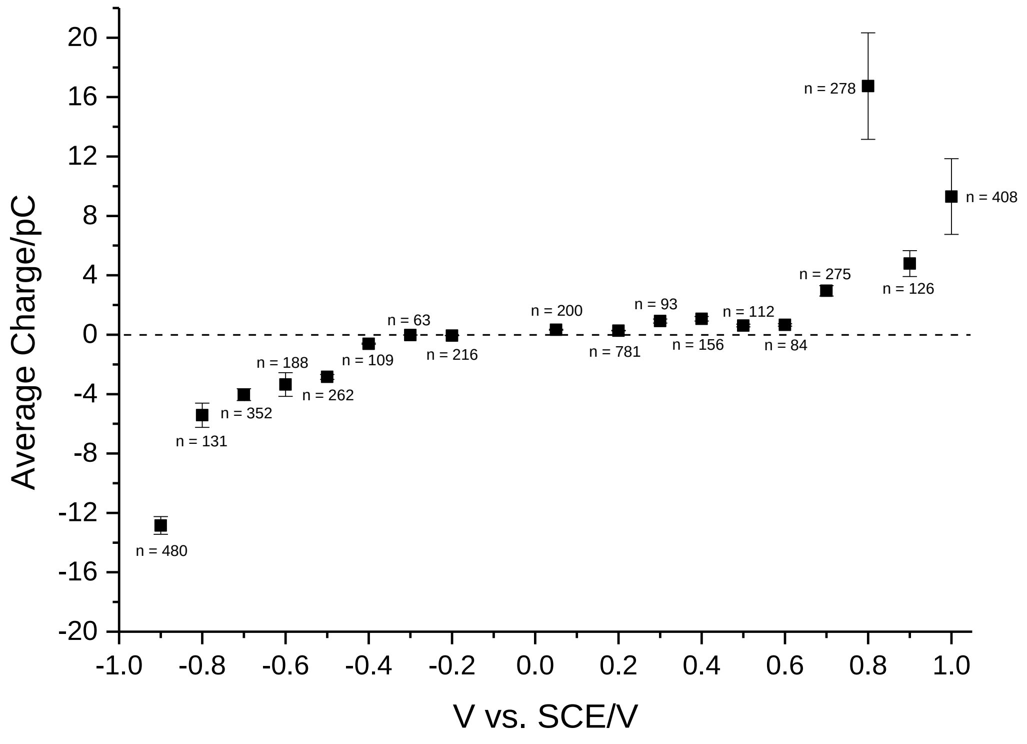

Figure S2: Plot of average impact charge vs. applied potential (vs. SCE) in 5.9 × 10^{-13} mol dm^{-3} suspension. The n numbers indicate the number of impacts involved in generating each data point and its error bars.

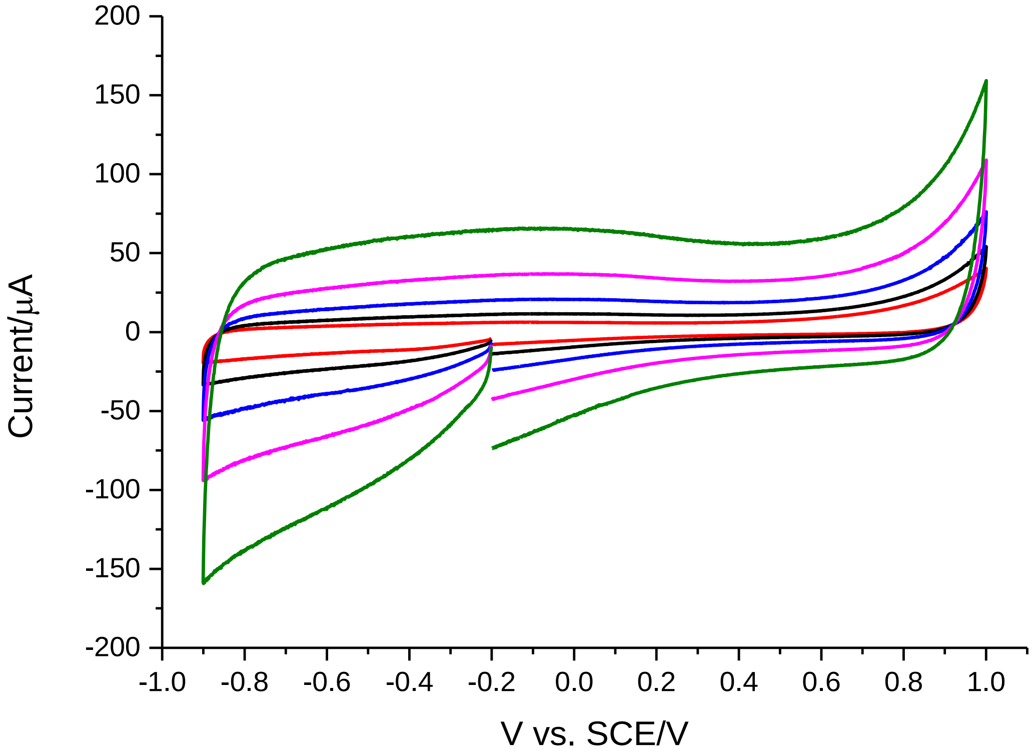

Figure S3: The voltammetric response of Graphene Nanoplatelets drop cast (7.9 μg) onto a glassy carbon electrode (BAS Technicol, USA, diameter 3 mm) in supporting electrolyte (0.1M KCl, 50 mM potassium monophosphate, 50 mM potassium diphosphate) recorded as a function of scan rates (25 mV s^{-1}, red line; 50 mV s^{-1}, black line; 100 mV s^{-1}, blue line; 200 mV s^{-1}, magenta line; 400 mV s^{-1}, green line).

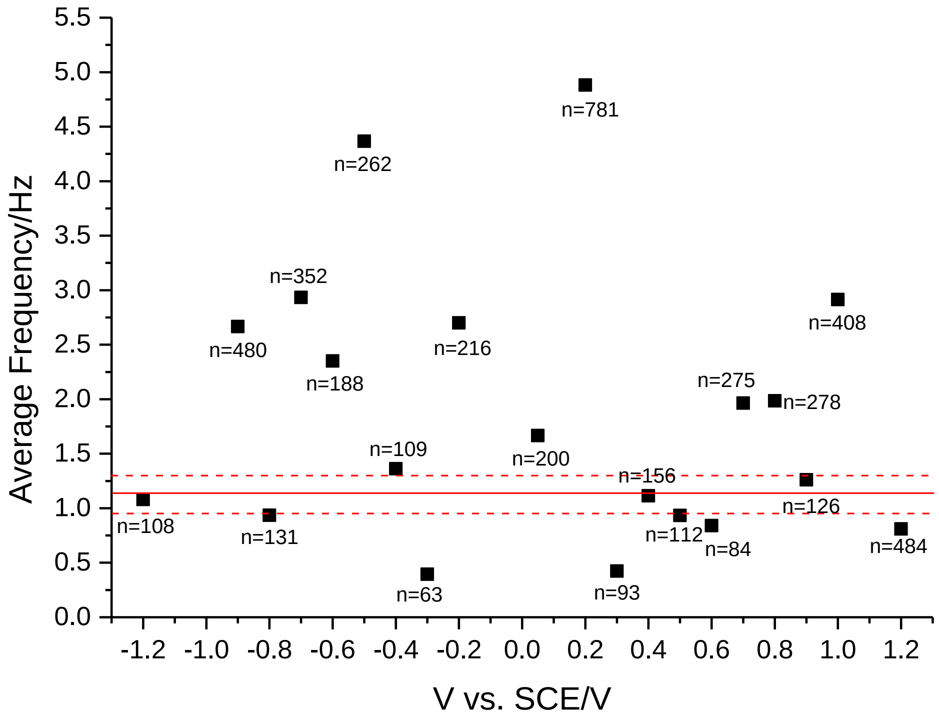

Figure S4: Plot of average impact frequency vs. potential applied (vs. SCE) in 5.91 × 10^{-13} mol dm^{-3} suspension. The wide scatter indicates a lack of frequency bias with respect to the potential applied. The numbers n indicate the number of impacts the dots plotted are based on.

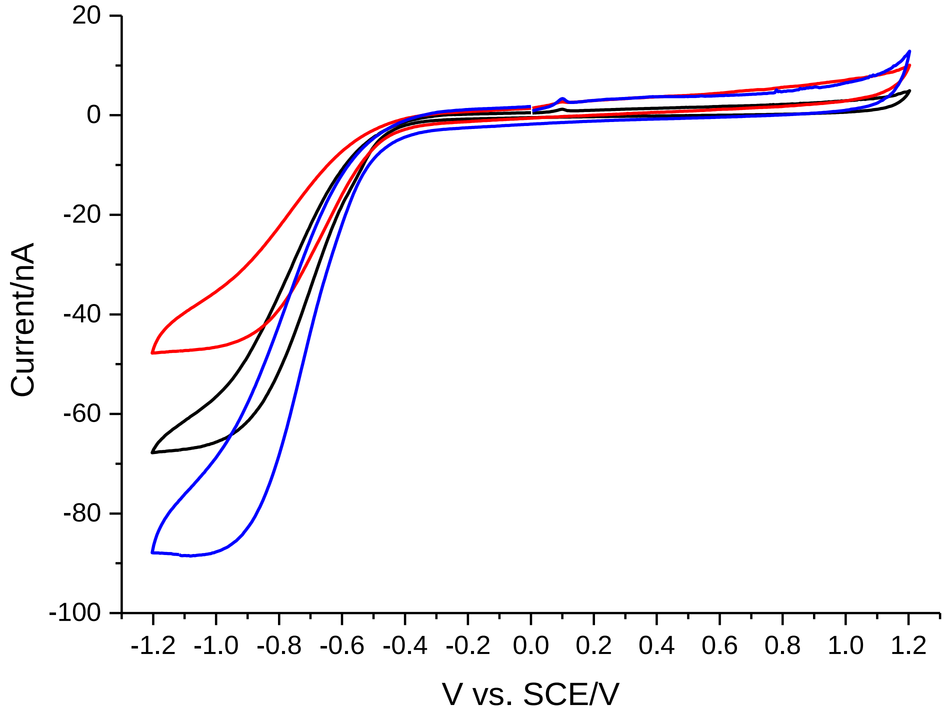

Figure S5: The voltammetric response of a cylindrical carbon fibre wire electrode in supporting electrolyte (0.1M KCl, 50 mM potassium monophosphate, 50 mM potassium diphosphate) recorded as a function of scan rates (25 mV s^{-1}, red line; 50 mV s^{-1}, black line; 100 mV s^{-1}, blue line). The reduction current likely indicates 50 µM of oxygen in the solution due to limitations of degassing.

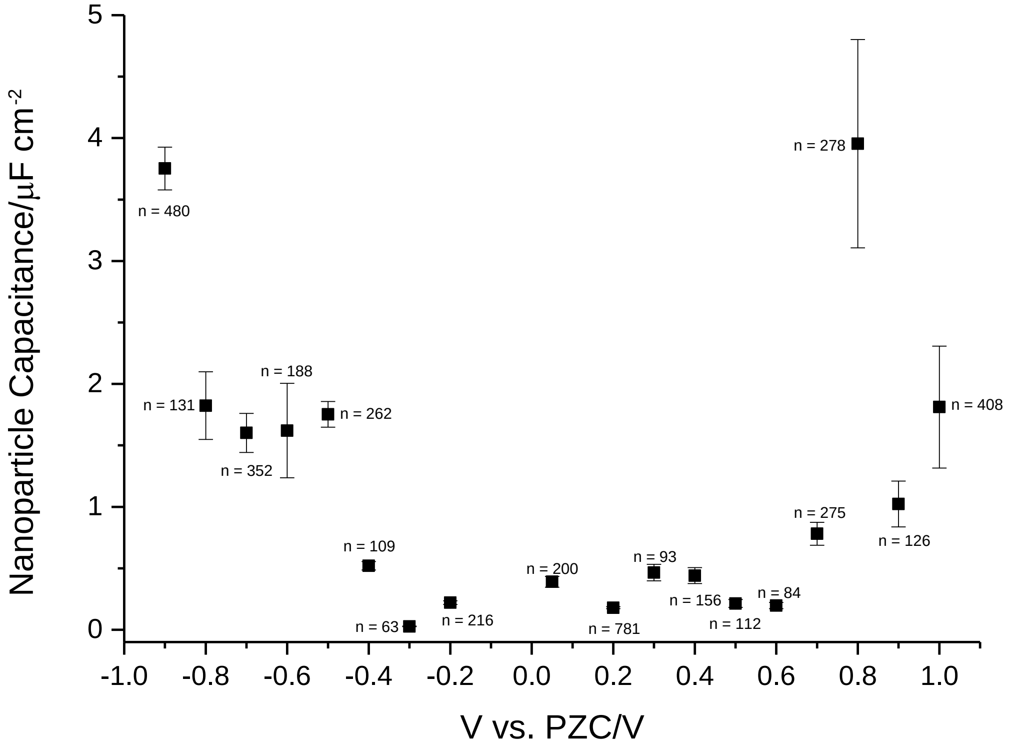

Figure S6: Plot of nanoparticle capacitance vs. PZC in 5.91 × 10^{-13} mol dm^{-3} suspension. The plot shows a non-linear relationship of potential (vs. PZC) to nanoparticle capacitance. The n numbers shows the number of impacts involved in generating each data point and error bars.
